# Sepsis as the Grand Mimic of Secondary Hemophagocytic Lymphohistiocytosis: *Serratia marcescens* Bacteremia with Concomitant Decompensated Cirrhotic Liver Disease

**DOI:** 10.1155/2023/9916937

**Published:** 2023-08-29

**Authors:** Anthony M. Lim, Jonathan R. Ghazaleh, Robert M. Cacdac, Julia K. Oberndorf, Marrey Ruby L. Quizon, Justin M. Thomas

**Affiliations:** Eisenhower Medical Center, 39000 Bob Hope Drive, Rancho Mirage, CA 92270, USA

## Abstract

Secondary hemophagocytic lymphohistiocytosis (HLH) is an elusive entity with sequelae that may be confused with sepsis. We discuss a 45-year-old man with decompensated liver cirrhosis with sepsis treated with broad-spectrum intravenous antibiotics. Further work-up initially supported sepsis-HLH overlap syndrome (SHLHOS) and corticosteroids were added. Ongoing refractory hypotension ensued, and the patient passed within 31 hours of presentation. Based on the patient's overwhelming immune activation and clinical course likely unsalvageable by cytotoxic immunosuppressive agents, the patient was diagnosed with sepsis with acute end organ dysfunction. This case report illustrates both the diagnostic challenge of sepsis versus HLH, which both require very different treatments, and the potential for rapid clinical decline without swift recognition and management of the true pathology.

## 1. Introduction

Hemophagocytic lymphohistiocytosis (HLH) can be either primary or secondary. The primary form of HLH is commonly seen in children and is due to familial/genetic causes, whereas underlying malignancies, rheumatological disorders, and infection (most commonly viral) can trigger secondary HLH [1]. Secondary HLH remains a very rare and an underrecognized entity especially in adults [2]. Interestingly, we have seen this entity emerge with the recent COVID-19 pandemic with around 100 cases reported [3]. The diagnosis, using HLH-2004 criteria, requires meeting ≥ 5 of 8 criteria: fever, organomegaly (hepatomegaly and splenomegaly), cytopenias ≥ 2 lineages, hypertriglyceridemia ≥ 3.0 mmol/L and/or hypofibrinogenemia ≤ 1.5 g/L, ferritin ≥500ug/L, low or no NK cell activity, soluble IL-2 receptor (s − IL2R or sCD25) ≥ 2400 U/mL, and biopsy-proven hemophagocytosis in bone marrow, spleen, or lymph nodes [4]. In one validation study of 101 patients, the 2004 HLH criteria had a sensitivity and specificity of 91.0% and 83.3%, respectively, with a PPV of 97.6% and an NPV of 55.6% [5].

HLH is likely underreported given its underrecognition and mimic of sepsis [2]. HLH must be suspected expeditiously, as mortality remains high and treatment comprises aggressive immunosuppression [3, 4]. However, this immunosuppressive regimen presents challenges in patients with bacteremia and underlying immunosuppression. We report the case of a 45-year-old man with decompensated liver cirrhosis with confirmed Gram-negative *Serratia marcescens* bacteremia who perhaps met a set of diagnostic criteria for HLH but was ultimately determined to be sepsis with acute end organ dysfunction.

## 2. Case Presentation

This is a 45-year-old man with decompensated liver cirrhosis due to chronic alcohol use, type 2 diabetes, and intravenous drug use. In the last 3 months, he had bacteremia of an unknown source associated with *Lacticaseibacillus* species.

The patient was allegedly well and a passenger in a car with his friend, when suddenly he became unresponsive. Approximately 10 minutes later, he was dropped off at the emergency department (ED). On arrival, he was unresponsive and pulseless in rhythm of pulseless electrical activity and undergoing active cardiopulmonary resuscitation (CPR). CPR continued, endotracheal intubation proceeded, and he achieved return of spontaneous circulation in 20 minutes.

Initial evaluation showed a temperature 29.3°C (84.7°F), heart rate 75 (sinus rhythm), respiratory rate 24 breaths per minute, blood pressure 135/94 mmHg (while started on 5 microgram/minute of norepinephrine), and Glasgow Coma Scale 3. Examination showed bilateral crackles heard diffusely throughout both lungs, positive fluid wave and flank dullness suggesting ascites, and bilateral 3+ pitting edema below the thighs.

Overall, labs (Table 1) showed profound electrolyte disturbance with metabolic acidemia (normal anion gap due to profound electrolyte disturbance, but notably elevated lactate). Liver function test derangements, cytopenias, and coagulopathy were likely attributed to both decompensated cirrhosis (MELD-Na score 36) [6] and sepsis with disseminated intravascular coagulation.

**Figure 1:**
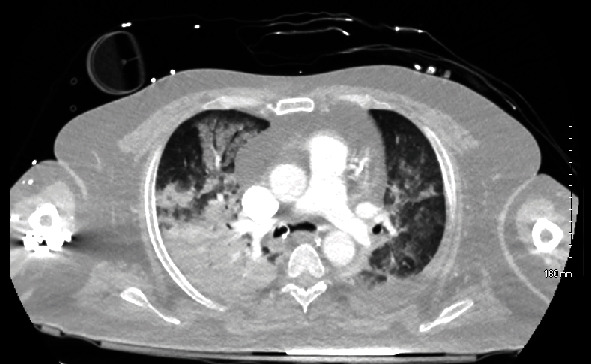
CT angiography chest abdomen pelvis showing extensive, heterogeneous, ground glass, and consolidative opacities throughout the lungs bilaterally, including dominant, dense consolidations in the right upper and right lower lobes. A small left pleural effusion is also noted.

**Figure 2:**
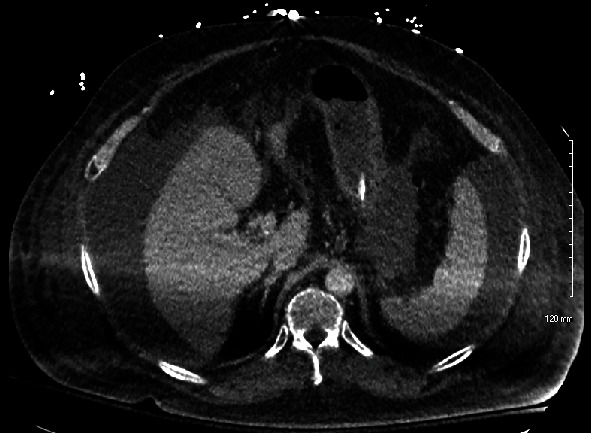
CT angiography chest abdomen pelvis showing radiographic splenomegaly, approximately 13 cm in the anteroposterior dimension.

CT angiography chest abdomen pelvis (Figures 1 and 2) showed extensive airspace opacities bilaterally concerning for a multifocal inflammatory process such as pneumonia, hepatosplenomegaly, and ascites and the endotracheal tube in the right mainstem bronchus (tube later moved to appropriate position).

ED management included IV fluid bolus 1500 mL normal saline and antibiotic cefepime. Hyperkalemia was treated with calcium gluconate. Vasopressor norepinephrine was titrated to maintain MAP ≥ 65 mmHg. He was admitted to the intensive care unit (ICU) for further management.

At ICU transfer, antibiotic regimen was changed to meropenem and vancomycin. Given hypothermia, thrombocytopenia < 50 K/*μ*L, coagulopathy, and relative hemodynamic instability, he did not undergo targeted temperature management [7].

A search for sepsis source was pursued. Paracentesis did not suggest spontaneous bacterial peritonitis (white blood cell count 81 cells per mm^3^). Ascitic fluid culture, urine culture, serum fungal (1-3)-beta-D-glucan assay, and respiratory panel PCR (influenza A/B, RSV, and SARS-CoV-2) were negative. Respiratory sputum culture showed few Staphylococcus aureus. One of four bottles of blood cultures grew Gram-negative bacilli Serratia marcescens resistant to ampicillin, sulbactam, and cefazolin.

As hypotension continued, he was given albumin, total 175 g 25% albumin, and 25 g 5% albumin. For concerns of bleeding, he was transfused total 4 units of red blood cells, 1 unit of platelets, and 10 units of cryoprecipitate.

Around 20 hours after presentation to ED, HLH was considered. Notably, the patient developed pancytopenia (white blood cell count was 1.1 K/*μ*L). Further work-up was sent: triglycerides, ferritin, and s-IL2R/sCD25. Triglycerides were not elevated, but ferritin was at 2994 ng/mL. After thorough consideration of immunosuppression in the setting of Gram-negative bacteremia, a decision was made to start noncytotoxic (versus a cytotoxic agent-like etoposide); hydrocortisone 100 mg q8h was started around hour 21; the patient received 200 mg hydrocortisone in total.

Unfortunately, hypotension persisted, even on hospital protocol's maximum doses of norepinephrine 30 microgram/minute and vasopressin 0.04 units/minute. Given ongoing concern of heart failure (brain natriuretic peptide > 5000 pg/mL), dobutamine was also started and titrated to 1.5 microgram/kg/min. Given guarded prognosis, the family changed the patient to “do not resuscitate.” 31 hours post-ED presentation, the patient went into asystole, did not receive CPR, and was pronounced expired. 48 hours postmortem, the sIL2-R/sCD25 level resulted elevated 7733.7 pg/mL (lab reference range 175.3–858.2 pg/mL). While the patient met the classic five criteria to diagnose HLH and could be considered for SHLHOS [4], given the rapid clinical course of sepsis with clear evidence of profound pathological immune activation and determination that cytotoxic immunosuppressive agents were of more risk than benefit, sepsis with acute end organ dysfunction was the most likely diagnosis.

## 3. Discussion

The distinction between HLH and overwhelming sepsis was challenging in our patient with liver cirrhosis. Liver cirrhosis is associated with both thrombocytopenia and splenomegaly [8], overlapping with two HLH diagnostic criteria, which were present in our patient. Meanwhile, patients with true HLH and acute liver failure may also present with fever, splenomegaly, and hyperferritinemia, also present in our patient [9]. The splenomegaly in our patient was only initially noted on the CT scan, while not certain it is more likely that this was associated with the patient's liver cirrhosis and portal hypertension, rather than as a new manifestation of suspected HLH. Our patient's lab results also seemed to support sepsis over HLH. Ferritin was 2994 ng/mL, which is less than the cutoff of marked elevation of ferritin > 3000 ng/mL (other cutoffs being 4000 up to 50,000 ng/mL) and thus could be explained by sepsis and liver disease [10], both present in our patient. s-IL2R/sCD25 was 7733.7 pg/mL, which is less than the cutoff of marked elevation of s − IL2R/sCD25 > 10,000 pg/mL, which again could be explained by sepsis [11].

Liver cirrhosis comprises both suppressive and immunosuppressive dysregulatory effects [12]. Our patient also had recent sepsis with an atypical organism (*Lacticaseibacillus* species), likely as a result of gut translocation [13]. Additionally, he has type 2 diabetes and consumes alcohol in excessive amounts, both associated with immunosuppression [14, 15]. His intravenous drug use increases his risk of sepsis [16]. Finally, the source of sepsis was likely thought to be from pneumonia, given the grossly abnormal CT angiography chest. Thus, there was strong evidence of classic sepsis, given the history and likely chronic pathological immunosuppression. Thus, in attempting to reconcile a diagnosis of HLH versus sepsis, one must question whether further cytotoxic immunosuppression would have been effective in this already highly immunosuppressed patient. Ultimately, it is unlikely that cytotoxic immunosuppressive agents would have benefited this patient, and so 200 mg of hydrocortisone was trialed, though in retrospect it is not likely that this was of benefit anyway.

Interestingly, the presence of both bacterial sepsis and HLH is rare, with fewer than five cases of HLH associated with Streptococcus pneumoniae infection [17] and one other case of HLH associated with Escherichia coli [18]. Should our patient have met more specific criteria above, e.g., with s − IL2R/sCD25 > 10,000 pg/mL, then perhaps cytotoxic immunosuppressive agents may have been considered. More likely overall, while the diagnostic criteria for HLH exist using the HLH-2004 standards [4, 5], perhaps it would be beneficial to remember the lessons of COVID-19 and the often-discussed “cytokine storm” phenomenon, which is a common manifestation of disseminated microbial infections [19]. In our patient at least, this was seen with confirmed Gram-negative *Serratia marcescens* bacteremia and sepsis with acute end organ dysfunction, with hyperferritinemia, elevated s-IL2R/sCD25, and progressively increasing serum creatinine and associated renal failure.

## 4. Conclusion

This case report showcases the great difficulty of distinguishing SHLHOS in patients who are likely immunosuppressed at baseline. The distinction is critical—SHLHOS requires cytotoxic immunosuppressive agents, while sepsis prohibits immunosuppressive treatment. The diagnostic criteria for HLH unfortunately are nonspecific if pertinent comorbidities including liver cirrhosis are seen in our patient. Thus, while perhaps clinicians should remain clinically astute and consider HLH, it remains tried and tested that “common things are common,” and sepsis with acute end organ dysfunction should more often remain the top differential.

## Figures and Tables

**Table 1:** Serum laboratory results throughout hospital course.

Laboratory test results	Reference range	Presentation (*t* = 0 hours)	*t*~9 hours	*t*~21 hours	*t*~30 hours
White blood cell (K/*μ*L)	3.8-10.8	5.9	1.1	5.6	6.7
Absolute neutrophil count (K/*μ*L)	1.9-10.8	2.9	0.7	3.8	5.6
Neutrophils (%)	51-89		61.9		84
Lymphocytes (%)	10-40		28.8		11.5
Hemoglobin (g/dL)	14-18	8.2	5.4	6.6	7.2
Platelet count (K/*μ*L)	150-450	36	51	33	39
Sodium (mmol/L)	136-145	122	124	130	127
Potassium (mmol/L)	3.6-5.1	6.2	4.3	4.6	5.5
Calcium (mg/dL)	8.6-10.3	7.2	7.7	8	7.7
HCO_3_^−^ (mmol/L)	23-29	12.6	9.5	12.8	11.3
Anion gap	4	11	11	12	15
Glucose (mg/dL)	70-105	236	129	79	81
Blood urea nitrogen (mg/dL)	7-25	37	38	39	41
Creatinine (mg/dL)	0.7-1.3	1.4	1.5	1.5	2.4
Albumin (g/dL)	3.5-5.7	<1.5	2	3.2	3
Bilirubin (mg/dL)	0.1-1.0	2.6	3	4.8	5.6
Alkaline phosphatase (IU/L)	34-104	130	70	82	50
AST (IU/L)	13-39	58	75	124	137
ALT (IU/L)	7-52	29	30	47	55
Ammonia (*μ*g/dL)	25-90	142			
Prothrombin time (seconds)	12.2-14.0	58.1		39.4	37.7
INR	0.9-1.1	6.5		4	3.8
aPTT (seconds)	25.9-32.8	188.6		82.8	74.3
Fibrinogen (mg/dL)	193-507	69	152		
Fibrin split products (*μ*g/mL)	<40	>40			
D-dimer (mg/L)	0-0.49	>20			
Brain natriuretic peptide (pg/mL)	1-100	520	551	3234	>5000
Triglycerides (mg/dL)	<150			26	
Ferritin (ng/mL)	30-400			2994	
Lactic acid (mmol/L)	0.5-2.2	5.2	6.7	6.5	
ABG pH	7.35-7.45	7.14		7.24	
ABG PaO_2_ (mmHg)	80-100	236		107	
ABG PaCO_2_ (mmHg)	35-45	28		23	
ABG HCO_3_^−^ (mmol/L)	20-26	10.4		12.5	

ABG = arterial blood gas; HLH = hemophagocytic lymphohistiocytosis. ~ to denote that time of lab collection is approximate, as many labs were collected at multiple times throughout the day. Presentation (*t* = 0 hours) laboratory results were the first set of serum investigations collected. Laboratory results at *t*~9 hours were the next set of routine labs, *t*~21 hours was around the time HLH was considered, and *t*~30 hours was 1 hour prior to death.

## Data Availability

The clinical and laboratory data used to support the findings of this study are available from the corresponding author upon request.
